# When Too Much Is Not Enough: Obsessive-Compulsive Disorder as a Pathology of Stopping, Rather than Starting

**DOI:** 10.1371/journal.pone.0030586

**Published:** 2012-01-26

**Authors:** Andrea L. Hinds, Erik Z. Woody, Michael Van Ameringen, Louis A. Schmidt, Henry Szechtman

**Affiliations:** 1 Department of Psychiatry and Behavioural Neurosciences, McMaster University, Hamilton, Ontario, Canada; 2 Department of Psychology, University of Waterloo, Waterloo, Ontario, Canada; 3 Department of Psychology, Neuroscience and Behaviour, McMaster University, Hamilton, Ontario, Canada; The University of Melbourne, Australia

## Abstract

**Background:**

In obsessive-compulsive disorder (OCD), individuals feel compelled to repeatedly perform security-related behaviors, even though these behaviours seem excessive and unwarranted to them. The present research investigated two alternative ways of explaining such behavior: (1) a dysfunction of activation—a starting problem—in which the level of excitation in response to stimuli suggesting potential danger is abnormally strong; versus (2) a dysfunction of termination—a stopping problem—in which the satiety-like process for shutting down security-related thoughts and actions is abnormally weak.

**Method:**

In two experiments, 70 patients with OCD (57 with washing compulsions, 13 with checking compulsions) and 72 controls were exposed to contamination cues—immersing a hand in wet diapers —and later allowed to wash their hands, first limited to 30 s and then for as long as desired. The intensity of activation of security motivation was measured objectively by change in respiratory sinus arrythmia. Subjective ratings (e.g., contamination) and behavioral measures (e.g., duration of hand washing) were also collected.

**Results:**

Compared to controls, OCD patients with washing compulsions did not differ significantly in their levels of initial activation to the threat of contamination; however, they were significantly less able to reduce this activation by engaging in the corrective behavior of hand-washing. Further, the deactivating effect of hand-washing in OCD patients with checking compulsions was similar to that for controls, indicating that the dysfunction of termination in OCD is specific to the patient's symptom profile.

**Conclusions:**

These results are the first to show that OCD is characterized by a reduced ability of security-related behavior to terminate motivation evoked by potential danger, rather than a heightened initial sensitivity to potential threat. They lend support to the security-motivation theory of OCD (Szechtman & Woody, 2004) and have important implications both for research into the biological mechanisms underlying OCD and for the development of new treatment approaches.

## Introduction

In obsessive-compulsive disorder (OCD), individuals feel compelled to perform certain security-related behaviors over and over again, such as washing their hands or checking that a door is locked, even though these repetitive behaviours typically seem excessive and unwarranted to them. Except for their intensity and persistence, these pathological acts closely resemble normal security-related behavior [1], [2]. Accordingly, Szechtman and Woody [3] proposed that OCD is a dysfunction of a biologically primal motivational system that normally helps to protect organisms from potential dangers like disease and attack by predators. Cues suggesting potential threat activate this Security Motivation System (SMS), which motivates preventative behaviors such as washing and checking, and the performance of these behaviors in turn typically provides negative feedback to terminate the security motivation. From this framework, OCD is basically a disorder in the regulation of a normal motivational system [4], [5], [6], [7], [8], [9], [10].

There are two different ways to explain the pathological intensity and persistence of security-related behavior in OCD [11]. One explanation posits that there is a dysfunction of activation—a **starting problem**—in which there is pathological intensity of excitation of the system by stimuli suggesting potential danger. According to this account, in OCD, threat concerns would be too readily or too intensely triggered by stimuli [12]. A contrasting explanation posits that underlying OCD is a dysfunction of termination—a **stopping problem**—in which there is failure of the normal process of termination of such security-related thoughts and actions. According to this account, in OCD, threat concerns may be elicited in the normal way, but once these concerns are activated, security-related behaviors are abnormally ineffective in turning these concerns off.

Building on the work of Reed [13], [14], we argued that characterizing OCD as a pathology of stopping better captures the behavioral profile of the disorder [3], [4]. When Reed analyzed the phenomenology of compulsive experiences in OCD, he found that the most common experience reported was an inability to stop—for instance, “I can't move on because I can't convince myself that I've finished what I'm doing” [14] (p.127). However, rather than conceptualizing OCD in terms of a general underlying cognitive disability to achieve closure, as Reed did, we posited that OCD results from the breakdown of a specific satiety-like mechanism by which engagement in security-related behavior normally shuts down the security motivation system. Phenomenologically, this stopping mechanism is associated with an internally generated satiety-like signal that serves as a terminator for the motivation. In individuals with OCD, performance of security-related behavior may fail to shut down activation of the system in the normal way. Such failure would explain why, in OCD, security motivation persists abnormally long and drives compulsive and obsessive behaviors.

In summary, OCD can be characterized either as a problem of starting (hypersensitivity to potential threat stimuli) or as a problem of stopping (dysfunction of a satiety-like, negative feedback mechanism). Here we report two experiments that shed light on these alternative conceptions.

### Respiratory Sinus Arrythmia as an Index of the Activation of Security Motivation

In these experiments, rather than relying only on subjective report, we employed respiratory sinus arrythmia (RSA) as an objective measure of the activation of security motivation. The rationale for using RSA stems from polyvagal theory [15], which posits a hierarchy of states of parasympathetic-sympathetic functioning. A state dominated by parasympathetic influence facilitates social behavior in circumstances that are safe from danger, whereas a state dominated by sympathetic influence facilitates fight-or-flight behavior in circumstances that present imminent danger. According to polyvagal theory, there is an intermediate state, occurring when attention is drawn to the environment because of potential threat or novelty, in which parasympathetic influence is reduced, so that the sympathetic system can be triggered quickly if it turns out to be required later.

This potential-threat state of autonomic function can be monitored by its characteristic effect on heart-rate variability. Heart rate varies in association with spontaneous breathing, and the degree of this variability, measured as RSA, reflects the influence of a vagal brake on the cardiac pacemaker, inhibiting the heart from beating at its intrinsically higher rate [15]. Removal of this brake makes the heart's inter-beat interval less modulated and hence more regular, yielding decreased RSA amplitude (measured in *ln msec* ^2^), which thus indicates a shift from a safe toward a potential-threat autonomic state. Further, RSA change is a relatively pure index of vagal brake removal, unlike heart rate which is also affected by other vagal and sympathetic factors.

## Experiment 1

Experiment 1 was designed to evaluate the following hypothesis: The problem in OCD is a reduced ability of security-related behavior to terminate motivation evoked by potential danger, rather than a heightened initial sensitivity to potential threat. In testing this hypothesis, the objectives of the experiment were: (a) to measure the sensitivity to activation by potential threat; (b) to measure the effectiveness of security-related behavior in terminating activation from potential threat; and (c) to determine whether either or both of those characteristics distinguish OCD patients from non-patient controls.

The paradigm used in this experiment was developed previously in a series of studies with non-patient participants [16]. These studies established that exposure to stimuli implying the threat of contamination activates security motivation, that this activation persists for a long time in the absence of corrective behavior, and that the corrective behavior of hand washing promptly deactivates the motivation. These studies also established that RSA change provides a sensitive objective index of the level of activation of security motivation [4], [16].

### Methods

#### Participants

Participants were 57 patients (16 men) and 57 non-patient controls (16 men). The patients were recruited through the McMaster University Medical Centre (Hamilton Health Sciences) (33 participants) or advertisements in the general community (24 participants). All patient participants had a primary diagnosis of OCD, with washing compulsions as the predominant symptom. Patients recruited through the clinic were diagnosed with either the Structured Clinical Interview for DSM-IV (SCID) [17] or the Mini-International Neuropsychiatric Interview (MINI) [18], and diagnostic status was confirmed by an experienced clinician (MVA). The Padua-R [19] was used to evaluate OCD symptom severity: All patients had total scores of at least 29, contamination subscale scores of at least 14, and a higher score on the contamination subscale than on the checking subscale. The non-patient control participants were recruited to match the age and gender distributions of the patients, but they reported no known mental disorders. The relative absence of OCD symptoms was confirmed using the Padua-R: All control participants had total scores of less than 29 and contamination and checking subscale scores of less than 14. Further details of the participants are provided in Table 1.

**Table 1:** Demographic and Clinical Characteristics of Participants in Experiments 1 and 2.

Characteristic	Experiment 1		Experiment 2	
	*OCD Patients* a ^,^ b	*Non-Patient Controls*	*OCD Patients* c ^,^ d	*Non-Patient Control*
Age (years)	33.95±9.38	31.16±7.53	29.62±6.78	29.80±6.38
Total Score on Padua-R	51.49±9.60	18.77±4.44	52.63±6.24	20.40±4.36
Contamination subscale, Padua-R	20.19±5.79	7.47±2.58	9.38±2.18	7.47±2.64
Checking subscale, Padua-R	9.49±3.18	7.25±2.71	8.20±2.70	24.38±3.71

Values are mean±SD.

a Comorbid diagnoses: None (70.2%), major depressive disorder (15.8%), generalized anxiety disorder (8.8%), alcohol abuse (7.9%).

b Psychotropic medications: None (21.1%), paroxetine or fluoxetine (35.1%), clonazepam (22.8%), citalopram or escitalopram (14.0%).

c Comorbid diagnoses: None (53.8%), major depressive disorder (23.1%), generalized anxiety disorder (23.1%).

d Psychotropic medications: None (15.4%), paroxetine or fluoxetine (38.5%), clonazepam (15.4%), citalopram or escitalopram (15.4%).

All participants were prescreened to ensure no known problems involving heart or lung function and no known allergies. Participants were asked to refrain from coffee and other stimulants for at least 2 hours prior to the study. The study was approved by the McMaster University and Hamilton Health Sciences Institutional Review Board. After a description of the study to the participants, written informed consent was obtained.

#### Materials and apparatus

Three levels of stimuli were designed to differ in the level of implied threat of contamination via germs or disease. The lowest level of threat was clean Styrofoam beads; the middle level was clean, dry diapers; and the highest level was wetted diapers. Previous research has demonstrated that with non-patient participants, contact with these stimuli elicit strongly differing levels of objective (RSA) and subjective response (rating of feelings of contamination), consistent with the implied level of contamination threat [15], [16], [20]. Participants were randomly assigned to the experimental conditions, subject to the constraint of comparable gender composition across groups.

For each of the two diaper conditions, seven of the diapers were piled in a hospital wastebin labeled *Pediatrics*; in the Styrofoam-beads condition, a similar but unlabeled container was used. Participants washed their hands at a sink fitted with an automatic, motion-activated faucet that was preset to deliver a consistent flow of warm water (at about 25°C).

For the measurement of RSA, the ECG signal was sampled at 2000 Hz and amplified using the Biopac data acquisition system. For each participant, a data file of interbeat intervals was analyzed to determine the RSA index of vagal tone, using the software CardioEdit and CardioBatch, according to the protocol developed by Porges and colleagues [15], [20].

#### Procedure

The participant remained seated throughout the experiment and was able to reach the sink at the required times by simply rotating the chair. The experimenter attached ECG electrodes and familiarized participants with operation of the automatic faucet and the procedure for hand washing. Participants were informed that at some point during the experiment, they would be asked to contact a stimulus that might or might not be contaminated. An initial 2-min, eyes-closed resting period allowed the collection of the baseline RSA. Next, participants made contact with the stimulus by submerging their left hand and arm into the bin containing the diapers or Styrofoam beads and moving their hand through the contents of the bin for 2 min. Participants were then instructed to remove their hand from the bin, hold it motionless in their lap (to avoid movement artifacts), and focus attention on it with eyes closed. At this time, the second 2-min period of RSA data was collected. Next, participants engaged in a prescribed 30-second period of hand washing. Specifically, they were told, “You have 30 seconds to wash, so if you are still washing after 30 seconds, I will ask you to stop.” A subsequent 2-min resting period with eyes closed allowed the collection of the third sample of RSA data. Finally, participants washed their hands freely for as long as they wanted. The subsequent 2-min resting period with eyes closed allowed the collection of the final sample of RSA data. At the end of each resting period, participants were asked to indicate their subjective level of contamination experienced during the last 2 min by pointing to the appropriate spot on a 15-cm line with the endpoints labeled “not at all contaminated” and “extremely contaminated.” Each response was quantified as the distance in centimeters from the lower endpoint. In addition, after both the fixed and free washes, participants likewise made a rating of their sense of satisfaction from their hand washing. For both the fixed and free washes, the amount of time in seconds that each participant actually spent washing was also measured. At the end of the study, all participants completed the Padua-R.

### Results and Discussion

The most important data in this experiment are the RSA values at three times of measurement: after contact with the stimulus (diapers or Styrofoam), after the fixed 30-second wash, and after the subsequent free wash. For each participant, these RSA values were subtracted from baseline RSA, so that higher scores represent higher levels of SMS activation.

The RSA change data were submitted to multivariate analysis of covariance (MANCOVA), with baseline RSA, age, and sex as covariates. The statistical tests for RSA change using baseline RSA as a covariate are identical to the corresponding tests for raw RSA with baseline RSA as a covariate; the advantage of using RSA change is simply to express decreases in RSA as increases in SMS activation. Statistical tests were based on Pillai's trace.

As indicated earlier, all the OCD-patient participants were actively symptomatic at the time of the study, despite any medication. Nonetheless, to examine possible effects of medication, an initial analysis of the OCD patients divided them into three groups by Medication Type: no medication (12 participants), antidepressants (32 participants), and clonazepam (9 participants). For the purpose of this analysis only, 4 OCD patients were excluded because they were taking a combination of both antidepressant medication and clonazepam. There were no significant effects of Medication Type on RSA, either as a main effect or in interaction with Stimulus (diapers or Styrofoam) and Time of Measurement. Hence, for subsequent analysis comparing the OCD patients to non-patient controls, we combined all the OCD patients (including the 4 patient participants on mixed medications) into one group.

**Figure 1:**
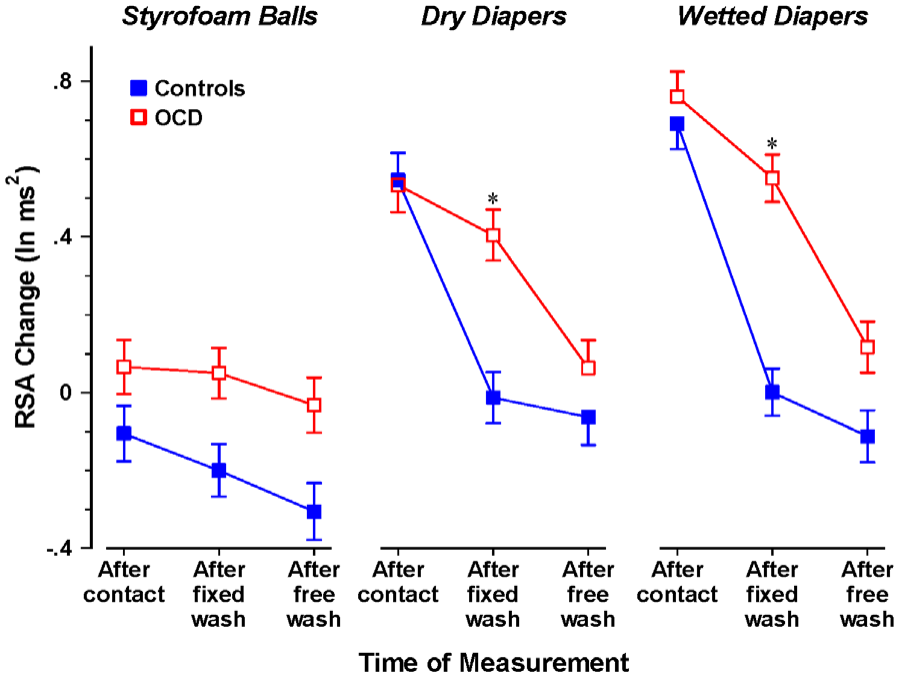
Effect of stimulus contact and subsequent hand washing on RSA in OCD patients with washing compulsions vs. non-patient controls. To index activation of the security motivation system, RSA change was computed as baseline RSA minus RSA level; thus, increases in the index correspond to greater activation Fixed wash was limited to 30 s; free wash was as long as the participant wanted. Note: * *p*<.05 vs. controls at the same time of measurement. Error bars represent 1 SE.

In the analysis of RSA, there were two statistically significant interactions with Group (OCD patients vs. controls): the three-way interaction of Group, Stimulus, and Time of Measurement, *F*(4, 210) = 4.48, *p*<.01, partial eta-squared = .08; and the two-way interaction of Group and Time of Measurement, *F*(2, 104) = 23.11, *p*<.001, partial eta-squared = .31. Figure 1 depicts the relevant adjusted means. Looking first at the after-contact time-of-measurement for each of the stimulus conditions, it was evident that for both the OCD patients and the control participants, contact with diapers elicited much greater activation than contact with Styrofoam. However, there was no significant difference between the OCD patients and the controls in this initial SMS response. This finding is inconsistent with the hypothesis, proposed by some theorists [12], that OCD stems from heightened sensitivity to activation by potential threats. Second, examining the effect of the fixed (30-second) wash in the two diaper conditions, this limited-time corrective behavior was significantly less effective in reducing SMS activity for the OCD patients compared to the controls. This finding supports our hypothesis that OCD stems from a reduced ability of security-related behavior to terminate motivation evoked by potential danger. Interestingly, after the free wash, when all participants had had the opportunity to wash as long as they wished, the difference between groups became smaller and not statistically significant, a result we interpret in the general discussion.

The corresponding MANCOVA on subjective ratings of contamination yielded a similar, but less striking pattern of results. The significant effect involving Group was the two-way interaction of Group and Time of Measurement, *F*(2, 105) = 12.48, *p*<.001, partial eta-squared = .19, reflecting the general tendency for feelings of contamination to drop after washing significantly less for OCD patients than for control participants. Consistent with the results for RSA, there were no significant differences between OCD patients and controls in their feelings of contamination immediately after contact with either dry or wet diapers; both groups rated their feeling of contamination similarly high.

**Figure 2:**
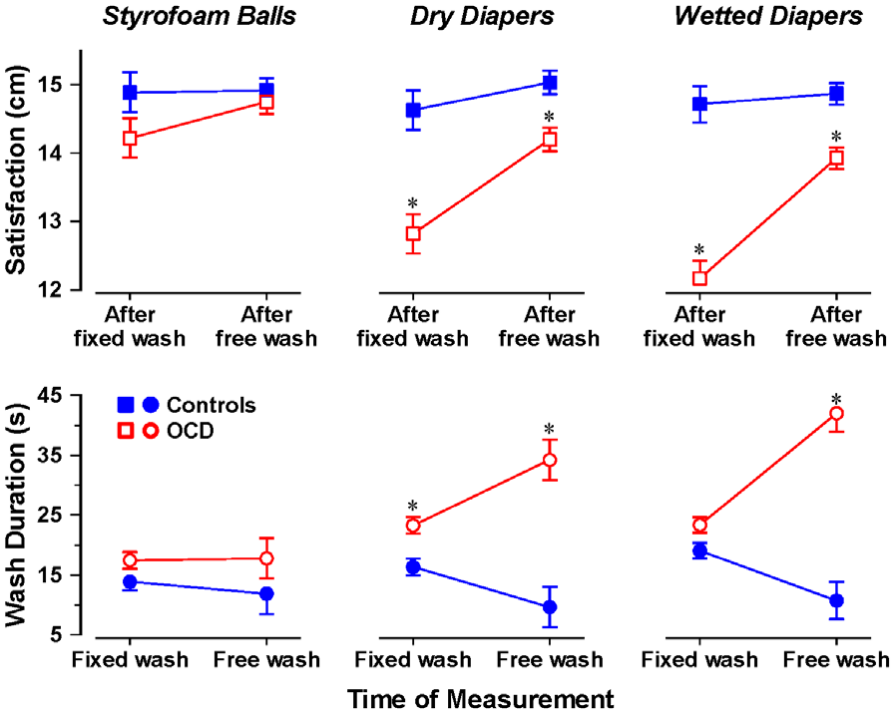
Effect of hand washing on satisfaction and wash duration in OCD patients with washing compulsions vs. non-patient controls. Note: * *p*<.05 vs. controls at the same time of measurement. Error bars represent 1 SE.

The results for actual wash duration and satisfaction clarify the overall picture. (For both of these outcomes, there were just two times of measurement: after the fixed wash, and after the free wash). A MANCOVA of wash duration with age and sex as covariates yielded two statistically significant interactions with Group: the three-way interaction of Group, Stimulus, and Time of Measurement, *F*(2, 106) = 8.34, *p*<.001, partial eta-squared = .14; and the two-way interaction of Group and Time of Measurement, *F*(1, 106) = 37.98, *p*<.001, partial eta-squared = .26. The corresponding MANCOVA of satisfaction yielded the same two significant effects: for Group×Simulus×Time, *F*(2, 106) = 3.13, *p*<.05, partial eta-squared = .06; and for Group×Time, *F*(1, 106) = 30.01, *p*<.001, partial eta-squared = .22. Figure 2 shows the adjusted means for these two variables. After exposure to both dry and wet diapers, the fixed wash left the OCD patients significantly less satisfied than the control participants. However, even though the OCD patients washed much longer than controls in the subsequent free-wash opportunity, their level of satisfaction with their hand-washing remained significantly lower than that of the controls. In short, even with substantially longer washing, the OCD patients did not achieve the level of satisfaction of the control participants. Overall, these satisfaction and wash-duration data, together with the RSA and contamination results, lend strong support to our hypothesis that OCD reflects a reduced ability of security-related behavior to terminate motivation evoked by potential danger, rather than a heightened initial sensitivity to potential threat.

A limitation of Experiment 1 is that it considered only one subtype of OCD, namely, washers. Thus, it is not clear whether the results apply to this subtype only, or would also apply to OCD patients with a different symptom subtype. Hence, Experiment 2 examines whether similar findings characterize OCD patients with checking compulsions.

## Experiment 2

A major issue in understanding OCD is to explain why it takes the form of distinct symptom clusters or subtypes that are reasonably stable over time, such as “washers” and “checkers” [21], [22], [23]. Why do OCD patients tend to have difficulty with only a particular domain of security concerns, but not all?

This question can be addressed in terms of the distinction between starting versus stopping mechanisms raised earlier. One possible explanation is that OCD patients have a specific starting problem, but no stopping problem at all, as argued by some investigators [12]. For example, an OCD washer would be hypersensitive to activation by threats of contamination, but not other potential threats. Another possible explanation is that all OCD patients have a general stopping deficit, but the domain in which they show symptoms is one in which they have high sensitivity to activation by a particular class of threat stimuli. A third possibility is that the stopping deficit in each OCD patient tends to be specific to a particular domain of potential threats. For example, a patient for whom washing is relatively ineffective in shutting down activation related to contamination threat may not have difficulty in terminating activation in response to other types of potential threats.

The foregoing experiment with OCD washers provided some evidence against the first two of these alternative possibilities, in that these patients did not show any indication of a starting problem in response to the threat of contamination. In addition, the results of that experiment lent support to the third possibility by showing a stopping deficit.

However, the design of Experiment 1 did not allow us to evaluate the specificity of this stopping deficit. In particular, would OCD patients whose predominant symptom is checking also show a stopping deficit in response to the symptom-unrelated threat of contamination? In Experiment 2, we used the basic paradigm of Experiment 1, with just the strongest of the three stimulus levels of implied threat (wet diapers), to compare OCD checkers to non-patient controls. If the stopping deficit in OCD is a general one, then the OCD checkers should differ from controls in the ways that OCD washers did in Experiment 1. Alternatively, if the stopping deficit is specific to the type of threat characteristic of the patient's symptoms, then the results for the OCD checkers should closely resemble those for the non-patient controls.

### Methods

#### Participants

Participants were 13 patients (4 men) and 15 non-patient controls (5 men). As in Experiment 1, the patients were recruited through the McMaster University Medical Centre, (Hamilton Health Sciences) (5 participants) or advertisements in the general community (8 participants). All patient participants had a primary diagnosis of OCD, with checking compulsions as the predominant symptom. OCD symptom severity was confirmed using the Padua-R: All patients had total scores of at least 39, checking subscale scores of at least 20, and a higher score on the checking subscale than on the contamination subscale. The non-patient control participants were recruited to match the age and gender distributions of the patients, but they reported no known mental disorders. The relative absence of OCD symptoms was confirmed using the Padua-R: All control participants had total scores of less than 29 and contamination and checking subscale scores of less than 14. Further details of the participants are provided in Table 1.

All participants were prescreened as in Experiment 1. This study was approved by the McMaster University and Hamilton Health Sciences Institutional Review Board.

#### Materials and apparatus

For all participants in Experiment 2, the potentially contaminated stimulus was the wet diapers, as used in Experiment 1. Materials and apparatus were as described for Experiment 1.

#### Procedure

RSA and subjective ratings were collected as described for Experiment 1.

### Results and Discussion

**Figure 3:**
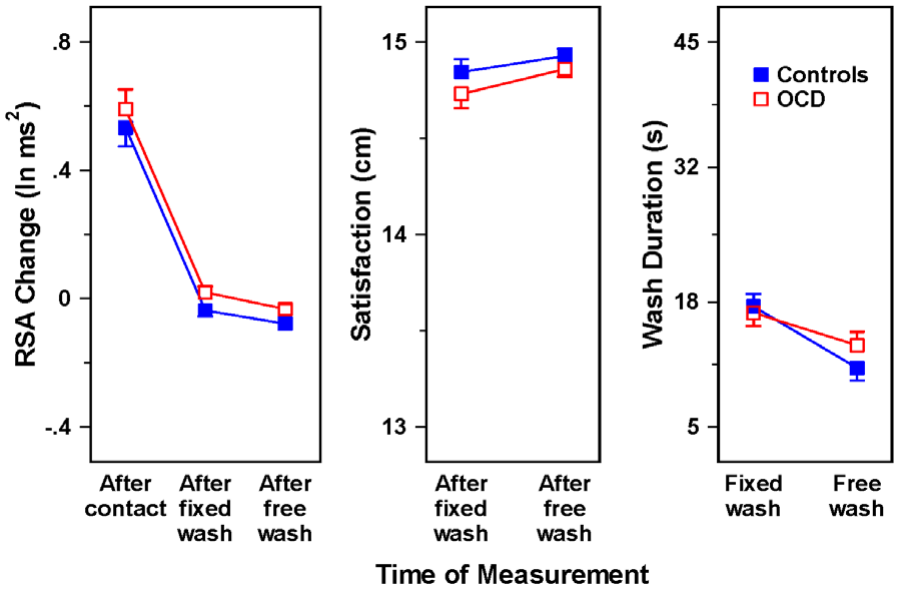
Effect of hand washing on RSA change, satisfaction, and wash duration in OCD patients with checking compulsions vs. non-patient controls. Note: Error bars represent 1 SE.

As for Experiment 1, the RSA change data were submitted to multivariate analysis of covariance (MANCOVA), with baseline RSA, age, and sex as covariates. Because in Experiment 2 there is just one level of stimulus potential threat, the most important effect is the two-way interaction of Group with Time of Measurement. This effect did not approach statistical significance, *F*(2, 22) = .10, *ns*. The leftmost panel of Figure 3 shows the relevant adjusted means, which are clearly virtually identical for the checking OCD patients and the non-patient controls.

The corresponding MANCOVAs for contamination, wash duration, and satisfaction also yielded Group×Time of Measurement interactions that did not approach significance: *F*(2, 23) = 1.13, *ns*; *F*(1, 24) = .55, *ns*; and *F*(1, 24) = 1.63, *ns*, respectively. The adjusted means for satisfaction and wash duration appear in the other two panels of Figure 3. For the OCD checkers and the non-patient controls in Experiment 2, the patterns of data closely resemble the patterns shown by the controls in Experiment 1, rather than the OCD washers in that experiment.

Thus, the corrective behavior of washing appears to be just as effective in alleviating SMS activation for OCD checkers as it is for controls. Taken together with the results of Experiment 1, these results from Experiment 2 support the hypothesis that the stopping deficit in OCD is specific to the type of potential threat that is characteristic of the patient's symptoms. Further support for this hypothesis would be provided by a future experiment that uses a checking paradigm to show that OCD checkers demonstrate a stopping deficit, but OCD washers do not.

## Discussion

The results of these experiments show that OCD reflects impairment in the ability of security-related behavior to terminate motivation evoked by potential danger, rather than a heightened initial sensitivity to potential threat. Our finding that OCD is not characterized by heightened initial sensitivity to potential threat is consistent with findings from other studies (e.g., [24], [25], [26], [27]). A second major finding from the present research is that the stopping impairment in OCD appears to be specific to the potential threats involved in the patient's symptoms, helping to explain why the disorder takes the form of relatively distinct symptom clusters. Such symptom specificity is also consistent with the findings of others (e.g., [28], [29]).

### Immediate versus Delayed Components to the Stop Signal

An unanticipated and intriguing finding from Experiment 1 with OCD washers was that after the free wash, when patients had washed as long as desired, the autonomic index of SMS activity (RSA change) indicated a deactivation of security motivation (consistent with their stopping of hand-washing). Yet the OCD patients reported that they did not feel as satisfied as controls that they had washed enough, suggesting incomplete deactivation of security motivation. Of several plausible interpretations for this finding, a particularly interesting one is a hypothesis that distinguishes immediate and delayed effects of a stop-signal deficiency in OCD.

There is much research on motivation to indicate that a “stop” or “satiety” signal that terminates an activated motivation is not an “all-or-none” event, but is rather a series of cascading mechanisms with distinct time lines. To illustrate, the behavioral act of eating brings hunger motivation to an end, but the mechanisms that ultimately arrest further eating and suppress hunger motivation are complex and distinct. For instance, filling the stomach with food distends the stomach and such stretching of stomach walls provides an immediate, albeit relatively short-acting, mechanical signal to inhibit the continuation of eating. Normally, this signal is of sufficient duration to allow the appearance of time-delayed signals as a result of postingestive metabolism of nutrients [30], [31], [32]. It is the effects of those metabolic factors on brain circuits that suppress the re-appearance of hunger motivation for an extended period of time [33], [34], [35], [36].

In a similar manner, we hypothesize that there are both immediate and delayed components to the stop signal that deactivates security motivation. Moreover, we propose that a deficiency of the delayed components makes OCD patients particularly susceptible to re-activation by potential threat. To provide an analogy, gastric loading with water rather than food can suppress hunger albeit briefly compared to a nutrient meal, and consequently the motivation to eat will emerge more quickly due to the absence of delayed metabolic inhibitory factors.

Recall that the OCD washers in our experiment showed a deficiency in generating the stop signal to deactivate security motivation, as measured by the inadequacy of the fixed 30-second hand-wash to restore the RSA change back to baseline. However, by extending the duration of hand-washing, the stop signal deficiency was partly ameliorated in a revealing way: Washing as long as desired returned the autonomic index of SMS activity back to baseline but not the subjective satisfaction with washing. Hence, we suggest, RSA change indexes the **immediate** component of the stop signal, while subjective satisfaction tracks the **delayed** or longer-lasting component of the stop mechanism. In other words, OCD patients can partially compensate for their deficient ‘stop’ mechanism by increasing the amount of corrective behavior, but this compensation does not generate the long-term “satiation” signal.

The essential prediction of the model above is that in OCD patients, the security motivation system, once activated, does not fully deactivate with corrective behavior, in that the system becomes sensitized to **re-activation** by potential threats. Note that the model predicts enhanced *re-activation* to potential threat stimuli, but normal activation upon first exposure (indeed, we found no initial hypersensitivity in our experiment with OCD washers).

### Possible Neural Mechanisms Underlying a Stopping Deficit

The present results are consistent with the work of other researchers investigating the idea that OCD stems from a stopping deficit of some kind [37], [38], [39], [40], [41], [42], [43], [44], [45], [46], [47], [48], [49]. However, because there are distinct types of “stopping” mechanisms in the literature [50], it is important to highlight that we theorize OCD pathology stems from one specific stop mechanism, namely, the negative feedback signal which normally terminates security motivation [3], [4], [11]. Importantly, this stop mechanism is distinctly different from the type of stop mechanisms proposed by several other investigators as impaired in OCD [51], [52], [53], [54], [55] and impulsivity [54], [56], [57], [58], [59], [60], [61]. Those authors propose that the pathological impairment is in “behavioral inhibition,” a form of fronto-executive control over motor responding. This type of cognitive control mechanism is evaluated by tests such as *stop-signal inhibition task*, *go/no-go task*, *delay-aversion/delay-discounting task*, and *the 5-choice serial reaction time task*, each paradigm tapping different components of the inhibitory control process [54], [56], [62], [63], [64], [65], [66]. Crucially, such “stop” tests examine if a deficit exists in the normal frontal mechanisms that can interrupt, cancel, or withhold, on-going or intended motor actions [56], [63]. In contrast, our theoretical model of the “stop” signal is aligned more with the motivational construct of “satiety,” and considers how the course of behavior aroused by a particular motivation comes to its normal end upon attainment of the goal object.

There are important implications of the present results for research into the biological mechanisms that underlie OCD. A consistent finding in brain imaging studies of OCD has been hyperactivity in the orbitofrontal-basal-ganglia circuit [67], [68], [69], suggesting that the OCD symptoms reflect pathology of over-activation in this network. However, if as indicated by the present results, OCD is due to a dysfunction of stopping, then the problem may not be this hyperactivity per se, but instead the dysfunction of neural pathways elsewhere that fail to turn off this activity. Hence, it would make sense to search for brain regions of hypoactivity in OCD, which may reflect dysfunctional negative-feedback, stopping pathways.

The neural origin of these stopping pathways is likely in the brainstem, as elaborated elsewhere [3], [4]. Such a supposition follows from the fact that the structure of security motivation is open-ended in that the external environment does not possess signals of goal-attainment—stimuli indicating that there is no potential danger. Hence, it is the actual performance of security-related behavior which signals goal-attainment and generates the negative feedback for terminating security motivation activity. These stop feedback pathways are proposed to innervate the limbic striatum and frontal cortex, and arrest the reverberant activity in the orbitofrontal-basal-ganglia circuit via serotonergic neurotransmission [3], [4]. Accordingly, possible brain regions of hypoactivity in OCD are neural sites elaborating proprioceptive feedback from the performance of species-typical security-related motor actions.

This emphasis on proprioceptive feedback mechanisms is consistent with the theory of motor control as active inference [70]. In this view, motor acts are driven by proprioceptive prediction errors, which are theorized to be evaluated at a low level in the motor hierarchy. In OCD, failure of proprioceptive sensory feedback to fulfill the predicted patterns of sensory signals could generate a mismatch or error signal, which in turn might interfere with the stop-feedback function of corrective actions.

### Possible Therapeutic Implications

The present results also have important therapeutic implications. First, although exposure with response prevention (ERP), the prevailing psychotherapeutic treatment for OCD, is quite effective, a sizeable proportion of patients do not comply with or find they cannot tolerate ERP. The perspective advanced here provides a different way of explaining to patients the underlying nature of their difficulties, which, by linking OCD to normal psychology, may connect better with their experiences, thus providing a more acceptable rationale for treatment and enhancing persistence with its difficult challenges. In particular, it may be explained to patients that OCD symptoms occur in response to cues that activate a normal, biologically primal motivational system that protects people from potential danger. However, security-related behaviors that for most people would readily shut down these primal concerns do not work well in people with OCD. Thus, OCD patients repeat these behaviors in an attempt to overcome their weak internal stop signal, which explains why “too much is not enough.” Leahy [71] has integrated this kind of explanation successfully into a CBT-based treatment protocol.

Second, complementing the emphasis in CBT on obsessive thoughts in OCD, the perspective advanced here highlights the role of compulsive behavior. Although compulsive responses are blocked in ERP treatment, recent research indicates that this is not necessary for treatment success; indeed, the opposite possibility, response intensification, may be as effective [72]. Thus, manipulation of compulsive behavior patterns and their negative-feedback function may offer new avenues for treatment. The basic idea would be to develop techniques to teach, or compensate for the weakness of, the security-satiety or stop signal. For example, some work suggests that hypnotic suggestions [73] and biofeedback [42], [43] can either block or attenuate confidence in internal signals. Hence, it is possible that such techniques can also be used to intensify or enhance the internal stop signal. Pharmacological interventions might also be developed for such enhancement.
